# Discussion on Dr. Brown’s Paper

**Published:** 1892-03

**Authors:** 


### DISCUSSION ON DR. BROWN'S PAPER *

---

S. S. AND G. ASSOCIATION.

---

DR. ROBERT T. MORRIS, New York: The cases in which gonorrhœa extends along the adjacent tracts are pretty well understood, at least their philosophy is known to us. But I know of no better field for new observation than that of the classification and elaboration of the cases of gonorrhœal septicæmia or gonorrhœal rheumatism, so-called. We can look forward to and expect all of the symptoms and complications from gonorrhœa following the mucous tracts, as described by Dr. Brown, but we cannot anticipate the results of gonorrhœal septicæmia. We may have different forms, from a simple sensitiveness of the nuchal cord to perhaps suppurative phlebitis of the pampiniform plexus, going on to pyæmia, and death from emboli or suppurative phlebitis. We have gradations, from a simple involvement of the fibrous tissue to death from septic embolism, and these cases are not well understood. Authorities do not describe them well. I am familiar with the literature, and there is really no satis-

* See page 607, December issue. This report received too late for preceding issue.—Ed.

factory literature upon the subject of systemic infection from the gonococci. I suppose Neisser's cocci do not in themselves produce suppuration, but are followed by the hungry camp followers, the pyogenic microbes, which devour whatever they want along the track of Neisser's gonococcus. There is hardly a physician in this room who has not to-day under his care some one case that he calls gonorrhœal rheumatism, which has gone on perhaps to hideous deformity. It may have been a simple urethritis at first, and if a diagnosis had been made early the disease might have flickered quickly. A case in point: A very wealthy gentleman had traveled all over Europe to be cured of rheumatism, and had been to all the springs and special institutions for rheumatism. When I saw him in New York I thought there was an element of septicæmia in the case. I found a stricture and post-urethritis. There was no difficulty in dividing the stricture and curing the post-urethritis, and the man is going about to-day without a stricture and without a single symptom of the rheumatism which he had been told was incurable by high authorities.

I repeat that there is no better chance for original work than in the elaboration and classification of the various cases of systemic gonorrhœal septicæmia.

One point in regard to peroxide of hydrogen. When we have chronic suppuration of the urethra or bladder, we can use peroxide of hydrogen in full strength in a certain proportion of cases without doing any harm. It must be warmed only a moment, and not very warm, before it is introduced into the bladder, as otherwise too much nascent oxygen would escape. One ounce of fifteen volume peroxide of hydrogen will throw off one pint of gas, so we can make this estimate when using peroxide in an obstructed bladder. The gas should be held in the bladder as long as possible, and then the patient passes it out as easily as he would pass water, with a sound of mockery for the old authorities who solemnly cautioned us against getting any sort of air or gas into the bladder.

DR. JOSEPH PRICE, Philadelphia: I agree with everything that has been said by Dr. Brown in regard to gonorrhœa. It is an exceedingly common and virulent disease. It fairly decimates the land. We perhaps see less of it as specialists, but the general surgeons have their wards and almshouses full of it. I consider it a virulent disease, one capable of very great mischief. The sequelæ of it are harmful. You all remember that a few years ago the treatment of gonorrhœa was prohibited in a large proportion of the general hospitals in large cities, and the disease was largely treated at one time by intelligent physicians, and the ravages of the disease were not so common then as at present. At present we all know that every drug-store is a clap shop, and it is exceptional for men or women to be treated by intelligent physicians.

I agree with what has been said in regard to the sequelæ of gonorrhœa. Schroeder and others have reported numerous cases in women, and we have all proved a number of times their observations and statements. I desire to call attention to one case in connection with the cases reported by Dr. Brown. I once heard Dr. Agnew relate a case of a prominent lumber merchant, a man with large business interests, who had a family of nine children. He visited Philadelphia and met some of his old friends which ended in a debauch. This man died in five days thereafter from prostatic (deep-seated) abscesses in one of our prominent hotels. This is one of many cases. One of my class-mates in the University of Pennsylvania died in five days from acute prostatitis. Others have been exceedingly ill. I recall at least three deaths from gonorrhœa in medical students. I might refer briefly to some of my early experiences in the treatment of venereal diseases. While a resident physician in the Old Philadelphia Dispensary, it was then the rule not to treat venereal diseases in the institution, but I asked as a special favor of the Secretary that I be permitted to treat all venereal diseases after hours, as I was anxious to have a little experience just then in that direction. Consent was given, and I soon built up a huge clinic for the treatment of venereal diseases. I call attention to this to verify my position in gynecology. I soon had a clinic that took me an hour and a half or two hours to manage. I had a large number of cases of gonorrhœa, chancroid, bubo, phimosis, prostatic trouble, vesical and renal troubles. I took my meals, my dinner and sometimes my breakfast with my brother. I had a room in the dispensary. My rule was to go to a restaurant near by to get lunch, and sometimes my breakfast if I had slept late. I soon discovered that it was impossible to dine at a single restaurant without being waited on by some one whom I was treating for bubo or gonorrhœa. I had to give up going to restaurants to take my meals. Some of these patients would salute me: "Hello, Doc, what will you have?" I had to go back to my brother's and take my meals with him. I call your attention to this matter to emphasize the fact, that I have since operated upon the wives of those men for huge pus tubes, ovarian abscesses, not by the dozen, but by the hundreds. I am speaking to prominent practitioners, men of large experience and good judgment. I am speaking from the standpoint of a specialist. I do nothing but pelvic and abdominal surgery, and do not consider me as explaining a point when making a statement of that character. For the last six years I have treated more cases of pelvic disease than any one else in Philadelphia.

Dr. Marcy treated many years ago at Harvard a young libertine for gonorrhœa. He had had many attacks, but was cured in Boston. He went home and prostituted a prominent young woman, and a few months later I operated on her for double ovarian abscess and double pyosalpinx, saving her life. I knew this young woman; I treated her family before she had her first sexual experience. I knew all about the case, knew when she contracted gonorrhæa, and from whom she got it, and I had the opportunity of watching her until I removed the lesions mentioned. I could allude, had time permitted, to a number of cases occurring in my own class-mates. I remember some prominent Union College graduates, one particularly in Albany, who contracted gonorrhœa. He went to a prominent house of prostitution and became infected. He studied theology at the Union Theological Seminary. He subsequently married a healthy woman, and she is still suffering from his contamination, and she remains sterile. I could cite a number of cases of this kind. I allude to these cases briefly, because I am convinced it would be an easy matter indeed to prove what has been said by Dr. Brown.

DR. GEO. A. BAXTER, Chattanooga, Tenn.: I take exception to two points in the paper. One is the impression left upon my mind of the perfectly satisfactory treatment the doctor gave his patients and the result. To me this point brought out by Dr. Price is a very strong one; that the majority of the mild cases are treated in the drug stores, and that those cases that reach the physicians are not by any means easy to cure; in fact, Dr. Price has more than emphasized the fact that they are not cured, otherwise years afterwards they would not be capable of infecting wives in subsequent years. I want to emphasize the point, that the majority of the severe cases are only partially cured, that the trouble is left behind, and if infection occurs it is from subsequent marriage. The disease, once removed, would be incapable of transmitting infection afterwards.

By medical treatment Dr. Brown promotes absorption of the stricture. I do not think any medical treatment will promote absorption of a stricture. I have never tried it. I have been under the impression that a stricture once formed was a permanent thing, unless relieved by electrical treatment or by cutting it. Even when dilated they are liable to recur and need actual surgical interference.

DR. A. VANDER VEER, Albany: Since I have been doing abdominal surgery quite exclusively for the past six or eight years, I can emphasize one point to which I desire to call your attention. When a conscientious man places himself under your care for the treatment of a specific urethritis, you treat him and he recovers, as you believe, he says to you: "Doctor, am I in a condition to marry?" I have that patient always go through this test: He comes to my office with his bladder containing six or eight hours of urine, and I allow him to pass all the urine he can, which I then have carefully precipitated. I examine it, and if there is any pus present or evidence of prostatic trouble, I say to him, you are not in a condition to marry.

DR. JOHN D. S. DAVIS, Birmingham: I fully agree with what Dr. Brown has said in regard to gonorrhœal infection. The real question for discussion is how to prevent the system from infection. A young man, several years ago, came under my care who had gonorrhœal infection that resulted in gonorrhœal rheumatism, which confined him to his bed for six months. He was finally able to go to work, and about eighteen months after that he received infection again and was again attacked with gonorrhœal rheumatism. He was in bed three months this time, and became very much alarmed from the stricture resulting from it. Last year he was inoculated again—I had obliterated the stricture in the meantime. He came to me in great anxiety and dread and pleaded, most woefully, for me to prevent or keep him, if possible, from having this rheumatism. I hardly knew what to do. I told the young man to go to bed and I would do all I could for him. I suggested to him that washing the urethra and bladder every three or four hours might prevent inoculation of the system. With this idea in view I made him an epicystic surgical fistula, by means of my first method, with trocar and canula, washed out his urethra and bladder with a fountain syringe every three hours, and in ten days he had no discharge. Urine was normal. I allowed the supra-pubic opening to close, but kept up the washing, and the young man got well without gonorrhœal rheumatism. The fistula was about five or six weeks in closing.

About six months afterwards another man came under my observation who had gonorrhœa with rheumatism following it. This was an old man who greatly feared the results that would come from rheumatism. I gave him my experience with the young man upon whom I had operated, and he asked me to do an operation. I did a similar operation upon him, and he had no rheumatism following it.

Now, the question is : Did I prevent, by this thorough and constant cleansing of the urethra, which can be done no other way, systemic infection? I am inclined to the opinion that I did. When opportunities present themselves I shall do the same thing again.

If, then, the above two cases were really cured by this perfect washing, is it not true, by this means, we have a power to prevent, in the male, all the ill effects emphasized in the doctor's paper—ureteritis, prostatitis, cystitis, rheumatism, etc? If so, what a saving of life and suffering is possible! I do not recommend that every one shall resort to this procedure, even in suitable cases, as much depends on the thoroughness of the cleansing and experience; tact and skill are required to keep urethra and bladder clean.

Dr. Bedford Brown, in closing the discussion, said: I am very much pleased that such eminent gentlemen as Drs. Morris, Price and others have indorsed my paper, and in regard to the difference of opinion of Dr. Baxter, I would say that probably he is laboring under a mistake in regard to my statement. I did not offer any method of treating gonorrhœa in this paper. Most of the cases did not come under my care during the early stages of gonorrhœa; I merely cited cases as evidence of constitutional or systemic infection following the disease. In my paper I placed on record those cases that have accumulated in my practice for a number of years, as evidences of systemic infection from gonorrhœal poisoning, and the treatment I have used was not intended for original or primary gonorrhœa at all. It was simply the treatment of the complications and constitutional conditions as they arose.

Now, in regard to promoting the absorption of a stricture by medical treatment. I am convinced that it has an effect; I do not think that it has a complete effect, but I am sure that it has some effect. I am sure what I say in regard to the treatment of the condition of the urine itself has a palliating effect on the progress of the stricture. The medical treatment has an effect over the irritation, the inflammation and engorgement of the stricture, and to a certain extent serves to promote absorption of the latter. I do not believe we can cure stricture by that method. I will say right here though, that my preceptor, Dr. B. W. Dudley, of Lexington, Kentucky, in whose office I spent three years, treated stricture alone by diet and medicine. I saw his cases; I studied them, I analyzed them, and I know that cases would come to him in which the sound would pass with the greatest difficulty, but after treatment the sound would enter the bladder without difficulty, and they would have no more trouble. His method was peculiar. He told me, and spoke of it in his lectures in the Transylvania University, that in his younger days a case of typhoid fever came under his charge, in which case there was a bad condition of stricture, one of the worst he had ever seen. The man was down with typhoid fever for three months; when he recovered from the fever the stricture was gone. The man was reduced to a skeleton. Dr. Dudley was a man of remarkable powers of observation and took his lesson from that case. From that time forward he treated strictures by diet in reducing the system and medicine with success, and I assure you I have seen him do it time and time again. But I do not pretend to cure stricture by alkaline treatment; I spoke of it as a means of relieving irritation and of promoting absorption of the inflammatory condition to a certain extent only. I have a case in point.

A young man, who had gonorrhœa seven to ten years ago, came under my care twelve months since for a bad case of stricture. The remarkable thing about this case was I could not introduce the sound without a most copious hemorrhage following. I do not know whether Dr. McGuire has ever had a case like that or not. Just as soon as the stricture was touched with the sound a violent hemorrhage would occur. It seemed as if some great artery had been severed. Well, I could not treat him mechanically. I commenced to treat him with alkalies, the citrate of potash and carbonate of potash, and kept him under the influence of that for four months. Now that man is comfortable. Before he was barely able to pass water. I used no mechanical treatment whatever. I cited this palliative treatment of stricture here to illustrate the influence exerted over inflammatory affections of the urinary tract by an alkaline treatment in maintaining a perfectly natural state of urine.

